# METTL3 Attenuates LPS-Induced Inflammatory Response in Macrophages via NF-*κ*B Signaling Pathway

**DOI:** 10.1155/2019/3120391

**Published:** 2019-10-24

**Authors:** Jinghua Wang, Shushan Yan, Hongying Lu, Shufeng Wang, Donghua Xu

**Affiliations:** ^1^Clinical Medicine College, Weifang Medical University, Weifang 261000, China; ^2^Department of Rheumatology, The Affiliated Hospital of Weifang Medical University, Weifang 261000, China; ^3^Department of Gastrointestinal and Anal Diseases Surgery, The Affiliated Hospital of Weifang Medical University, Weifang 261000, China; ^4^Functional Laboratory, Clinical Medicine College, Weifang Medical University, Weifang 261000, China; ^5^Medical Experimental Training Center, Weifang Medical University, Weifang 261000, China

## Abstract

Methyltransferase-like 3 (METTL3), an RNA N^6^-methyladenosine (m^6^A) methyltransferase, is essential for the m^6^A mRNA modification. As a key enzyme of m^6^A methylation modification, METTL3 has been implicated in immune and inflammation regulation. However, little is known of the role and underlying mechanism of METTL3 in rheumatoid arthritis (RA). The aim of the present study is to elucidate the function and potential mechanism of METTL3 in RA pathogenesis. We used quantitative real-time polymerase chain reaction to detect the expression of METTL3 in RA patients and controls as well as the macrophage cell line. CCK-8 was used for cell proliferation assay. Enzyme-linked immunosorbent assay (ELISA) was adopted to estimate the generation of IL-6 and TNF-*α* in macrophages. Western blot and immunofluorescence were applied to evaluate the activation of NF-*κ*B in macrophages. The expression of METTL3 was significantly elevated in patients with RA. It was positively associated with CRP and ESR, two common markers for RA disease activity. Besides, LPS could enhance the expression and biological activity of METTL3 in macrophages, while overexpression of METTL3 significantly attenuated the inflammatory response induced by LPS in macrophages. Moreover, the effect of METTL3 on LPS-induced inflammation in macrophages was dependent on NF-*κ*B. This study firstly demonstrates the critical role of METTL3 in RA, which provides novel insights into recognizing the pathogenesis of RA and a promising biomarker for RA.

## 1. Introduction

Rheumatoid arthritis (RA) is an autoimmune disease with chronic and reduplicated joint destruction, which is a highly disabling disease because of joint deformity and loss of function [1]. Joint inflammation can cause redness, swelling, pain, and joint deformity [2]. The etiology of RA is still largely unknown. It has been well established that the development of RA is attributed to genetic and environmental factors, such as tobacco smoking [3]. Apart from those, obesity, stress, nervous depression, and female hormones play vital roles in the pathogenesis of RA [3]. Many studies have suggested that dysregulation of the immune system, including abnormal activation and T and B lymphocytes, mast cells, and macrophages, is closely associated with the development of RA [4]. High levels of autoantibodies generated by dysregulated B cells can cause lung injuries, such as anticitrullinated protein antibodies [5, 6]. Inflammatory cells can be attracted and recruited to inflammatory sites under stimulation of considerable mediators from macrophages and mast cells. Thus, sustained and chronic inflammation leads to joint injuries and deformity [1]. As a result, it is essential to identify novel and promising biomarkers for the early diagnosis and targeted therapy of RA patients.

During the past few years, the role of N^6^-methyladenosine (m^6^A) methylation of RNA in autoimmunity, inflammation, and cancer has drawn close attention [7–10]. m^6^A is the most common posttranscription modification of RNA [11]. The key enzymes for m^6^A methylation modification primarily include m^6^A methyltransferase (writer), m^6^A demethylase (eraser), and m^6^A RNA-binding proteins (reader) [12]. Methyltransferase-like 3 (METTL3) is a key enzyme of m^6^A methylation modification and an important member of the methyltransferase complex including METTL3, METTL4, and Wilms tumor 1-associated protein (WTAP) [13]. It has been demonstrated that m^6^A methylation mediated by METTL3 has tissue and cell specificity [12–15]. Therefore, the role of METTL3 may alter in different tissues and cells [16]. METTL3 has been previously reported as a tumor suppressor by upregulating the m6A modification of genes [17]. However, Chen and colleagues have found that METTL3 could promote liver cancer progression by posttranscriptional silencing of SOCS2 via YTHDF2 [18]. More interestingly, the study by Feng et al. has implicated that METTL3 could inhibit inflammation by affecting the alternative splicing of MyD88 [19]. Nevertheless, the role of METTL3 in RA, an autoimmune and inflammatory disease, remains vague up to date. In the current study, we investigate the expression of METTL3 in RA and its relationship with disease activity. A series of cellular experiments in vitro have been performed to elucidate the potential role of METTL3 and its molecular mechanisms in RA, which may help to explore the pathogenesis of RA and explore novel biomarkers for RA.

## 2. Materials and Methods

### 2.1. Participants

Patients (47) who participated in this study were admitted to the affiliated hospital of Weifang Medical University from March 2018 to September 2018. 30 controls registered in the same hospital at the same time for health examination, while controls with a history of rheumatoid diseases were all excluded. All RA cases were new active patients and had not yet been treated with disease-modifying antirheumatic drugs and/or steroids before blood sample collection. Those RA patients accompanied by other rheumatoid diseases were excluded, such as Sjogren's disease. The present study was approved by the Institutional Ethics Committee of our hospital. Patients and controls had all signed the written informed consent before tests. Characteristics of all participants are presented in detail in Table 1.

### 2.2. Cell Culture and Transfection

THP-1 cells were cultured in RPMI1640 culture medium (Invitrogen Corp., Grand Island, NYUSA) administrated with 10% fetal bovine serum (Gibco, Carlsbad, CA, USA) plus penicillin, streptomycin, and L-glutamine under 5% CO_2_ at 37°C. Before treatment, THP-1 cells were stimulated and induced to macrophage-like cells (pTHP-1) by 100 ng/ml phorbol-12-myristate-13 acetate (PMA, Sigma, USA) for 48 h. Cells were transfected by lentivirus plasmid with polybrene (8 *μ*g/ml) for 48 h and used for the following experiments.

### 2.3. Quantitative Real-Time Polymerase Chain Reaction (qRT-PCR)

Peripheral blood mononuclear cells (PBMCs) were extracted from fresh blood samples by Ficoll-Paque lymphocyte isolate reagent (TBD, Tianjin, China), which were centrifuged at 2000 rpm for 30 min. We extracted peripheral blood monocytes using CD14 microbeads (Miltenyi Biotec, San Diego, CA) based on the protocol. Total RNAs were extracted from these cells by TRIzol (Invitrogen, USA) and quantified by measuring the absorbance with the UV spectrophotometer. A total of 1 *μ*g RNA was used to synthesize cDNA, which was used as a template for PCR by use of a SYBR Green Mastermix kit (Takara, Dalian, China). Primers were shown as follows. GAPDH: forward primer: GCACCGTCAAGGCTGAGAAC; reverse primer: GGATCTCGCTCCTGGAAGATG. METTL3: forward primer: TTGTCTCCAACCTTCCGTAGT; reverse primer: CCAGATCAGAGAGGTGGTGTAG. METTL14: forward primer: AGTGCCGACAGCATTGGTG; reverse primer: GGAGCAGAGGTATCATAGGAAGC. Obesity-associated protein (FTO): forward primer: AGAGCTCTAGAACCACCATGGATTACAAAGATGAC; reverse primer: CTAAGATTGCGGCCGCCTAGGGTTTTGCTTCCAGAAGC. AlkB homologue 5 (ALKBH5): forward primer: CGGCGAAGGCTACACTTACG; reverse primer: CCACCAGCTTTTGGATCACCA. YTHGF1: forward primer: ACCTGTCCAGCTATTACCCG; reverse primer: TGGTGAGGTATGGAATCGGAG. YTHGF2: forward primer: AGCCCCACTTCCTACCAGATG; reverse primer: TGAGAACTGTTATTTCCCCATGC. IL-6: forward primer: AGTCCTGATCCAGTTCCTGC; reverse primer: CTACATTTGCCGAAGAGCCC. TNF-*α*: forward primer: ATGTGGCAAGAGATGGGGAA; reverse primer: CTCACACCCCACATCTGTCT.

### 2.4. Total m^6^A Measurement

Cells were seeded in six-well culture dishes overnight in serum-free RPMI1640 culture medium. Then, cells were treated with 1 *μ*g/ml LPS (Sigma, CA, USA) for 0, 6, 12, and 24 hrs. RNAs were extracted according to the protocol. The kit for methylation quantification (EpiGentek, Farmingdale, NY, USA) was applied to determine levels of m^6^A RNA modification in cells.

### 2.5. Cell Counting Kit (CCK-8)

We used CCK-8 kit (Vazyme Biotech, Nanjing, China) to determine cell proliferation. In brief, 2 × 10^4^ per well THP-1 cells were incubated and activated by PMA (100 ng/ml) for 48 hrs in a 96-well plate and cultured with serum-free culture medium for 12 hrs and then stimulated by 1 *μ*g/ml LPS (Sigma, USA) for 0, 12, 24, and 48 hrs. The absorption at 450 nm was determined by the microplate reader (BioTek, USA) after incubation with CCK-8 reagent for 2 hrs at 37°C.

### 2.6. Enzyme-Linked Immunosorbent Assay (ELISA)

The concentrations of IL-6, TNF-*α*, ESR, and CRP were determined by ELISA according to the kit's protocol (R&D Systems, USA). We determined the absorption at the wavelength of 450 nm with a correction wavelength of 540 nm. Experiments were repeated for three times.

### 2.7. Western Blot

A total of 30 *μ*g/channel proteins were used for detection extracted from peripheral blood monocytes and pTHP-1 cells. We adopted the Bradford assay kit (Bio-Rad Laboratories, CA, USA) to quantify proteins. Protein samples were incubated with antibodies of METTL3 (Cell Signaling Technology, USA) and p-NF-*κ*B (Cell Signaling Technology, USA), and *β*-actin (Sigma, USA).

### 2.8. Immunofluorescence

After being activated by LPS (1 *μ*g/ml) for 4 hrs, the status of phosphorylated NF-*κ*B in the nuclear of pTHP-1 cells was estimated by immunofluorescence. Briefly, pTHP-1 cells were incubated with p-NF-*κ*B antibody (CST, USA) and then the secondary antibodies labelled with FITC. Finally, cells were detected using a Confocal Laser Scanning Microscope (Leica TCS SP8).

### 2.9. Statistical Analysis

Statistical analysis was carried out by use of GraphPad Prism (GraphPad Software, CA, USA). Data used for analysis was normal distributed. Unpaired Student's *t*-test or one-way ANOVA was used for statistical analysis. Pearson correlation analysis was used when analyzing the association between the expression of METTL3 and levels of ESR and CRP in serum. Two-tailed *P* < 0.05 was regarded to be statistically significant.

## 3. Results

### 3.1. Expression of RNA N6 Methylation-Related Genes in RA

**Figure 1:**
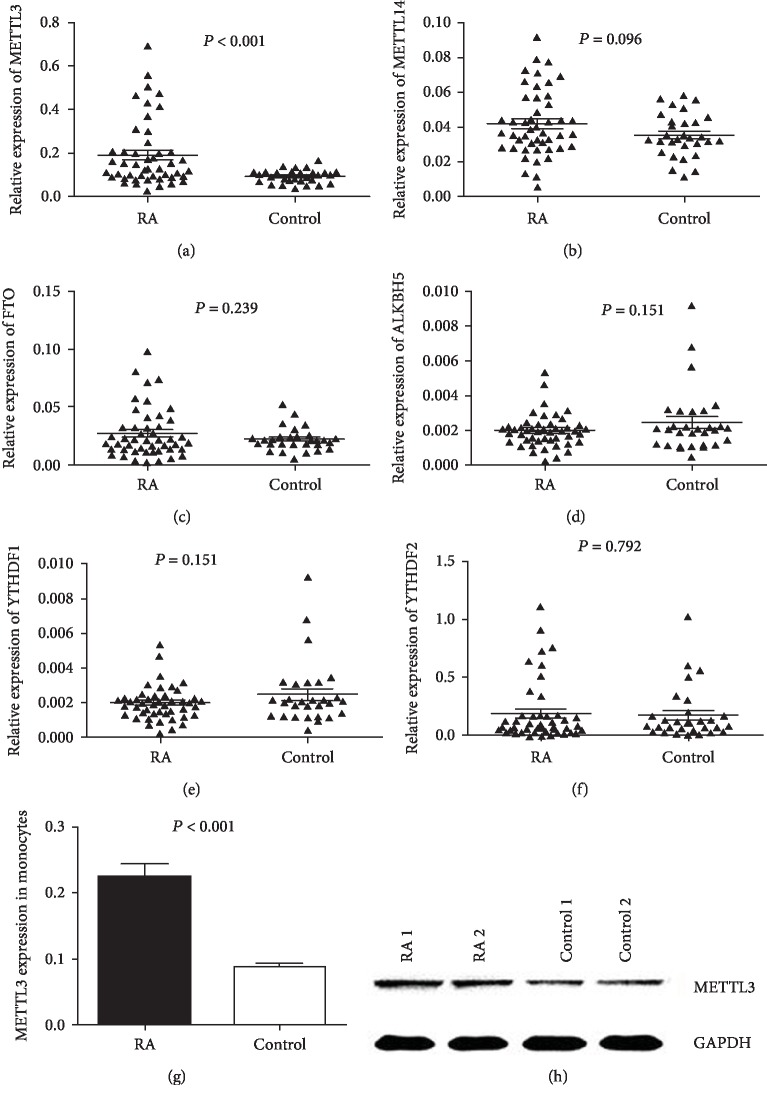
Expression of m^6^A methylation-related genes in RA (cases/controls: 47/30). (a) Increased mRNA level of METTL3 in RA in contrast to healthy controls. (b) mRNA level of METTL14 in RA when comparing with healthy controls. (c) mRNA level of FTO in RA compared with healthy controls. (d) mRNA level of ALKBH5 in RA in contrast to healthy controls. (e) mRNA level of YTHDF1 in RA when comparing with healthy controls. (f) mRNA level of YTHDF2 in RA in contrast to healthy controls. (g) Increased mRNA level of METTL3 in monocytes of RA patients in contrast to controls. (h) Increased METTL3 protein in monocytes of RA patients in contrast to controls.

We screened the expression of m^6^A methylation-related genes in the PBMCs of patients with RA and healthy controls by quantitative real-time PCR, including genes of METTL3, METTL14, FTO, ALKBH5, YTHDF1, and YTHDF2. As shown in Figure 1(a)–1(f), compared with normal controls, the expression of METTL3 was significantly increased in PBMCs from RA patients, while no difference was observed with regard to other key m^6^A methylation-related enzymes (METTL14, FTO, ALKBH5, YTHDF1, and YTHDF2). Monocytes were the main cells involved in inflammation and immune regulations. Here, elevated expression of METTL3 was also found in monocytes of RA patients in contrast to controls (Figures 1(g) and 1(h)). Taken together, METTL3 was upregulated in RA.

### 3.2. Association between METTL3 and Disease Activity of RA Patients

**Figure 2:**
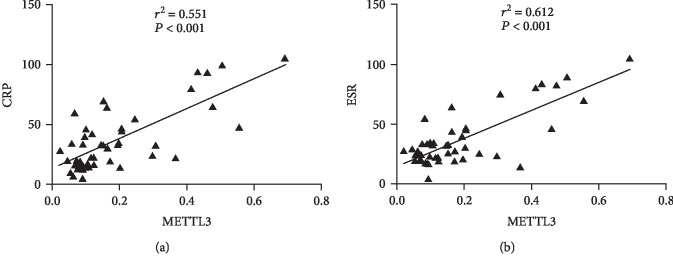
Association between METTL3 and RA disease activity. (a) Positive association of METTL3 with CRP in RA. (b) Positive association of METTL3 with ESR in RA.

Interestingly, Pearson correlation analysis showed that the expression of METTL3 was positively associated with CRP (Figure 2(a)). Similarly, positive association of the expression of METTL3 with ESR was observed in RA (Figure 2(b)). Accordingly, the elevated level of METTL3 in PBMCs might predict high disease activity of patients with RA.

### 3.3. LPS Enhanced the Expression of METTL3

**Figure 3:**
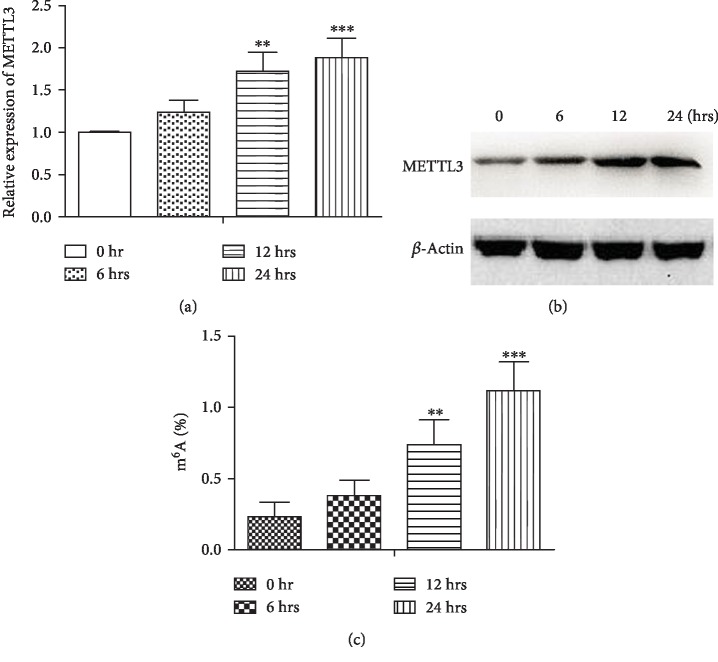
LPS promoted the expression and activity of METTL3 in pTHP-1 cells. (a) Increased mRNA level of METTL3 in cells (*n* = 3; LPS stimulation for 0 hr, 6 hrs, 12 hrs, and 24 hrs; ^∗∗^*P* < 0.01 and ^∗∗∗^*P* < 0.001). (b) Increased expression of METTL3 protein in cells (*n* = 3; LPS stimulation for 0 hr, 6 hrs, 12 hrs, and 24 hrs). (c) Total m^6^A content in cells (LPS stimulation for 0 hr, 6 hrs, 12 hrs, and 24 hrs; *n* = 3; ^∗∗^*P* < 0.01 and ^∗∗∗^*P* < 0.001).

Given the positive association between METTL3 and CRP as well as ESR in RA, we hypothesized that a high level of METTL3 could be induced in inflammatory conditions, which could thus defend against inflammation. As a result, we performed cellular experiments in vitro to observe the influence of inflammation on METTL3 expression and the level of m^6^A RNA modification in pTHP-1 macrophages. LPS could enhance the expression of METTL3 at both levels of mRNA and protein in a time-dependent manner (Figures 3(a) and 3(b)). Levels of m^6^A RNA modification were also increased in pTHP-1 macrophages in a time-dependent manner (Figure 3(c)). Taken together, inflammation could promote the expression and biological activity of METTL3. However, whether METTL3 affected inflammation in macrophages and RA development remained unknown.

### 3.4. METTL3 Inhibited the Activation of pTHP-1 Macrophages

**Figure 4:**
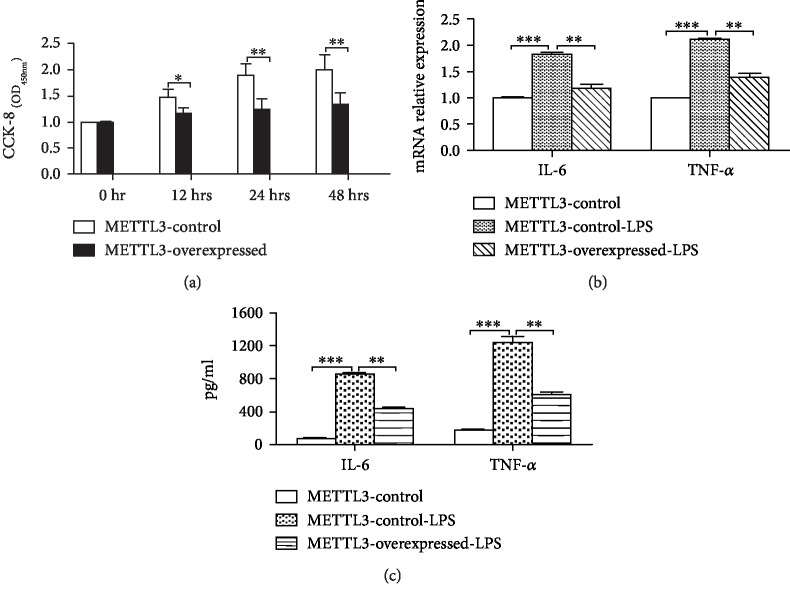
METTL3 inhibited the proliferation and activation of pTHP-1 cells. (a) CCK-8 assay detecting the proliferation of cells at 0 hr, 12 hrs, 24 hrs, and 48 hrs (*n* = 3; ^∗^*P* < 0.05; ^∗∗^*P* < 0.01). (b) Decreased mRNA levels of IL-6 and TNF-*α* in METTL3-overexpressed pTHP-1 cells (*n* = 3; ^∗∗^*P* < 0.01; ^∗∗∗^*P* < 0.001). (c) Decreased levels of IL-6 and TNF-*α* protein in the cultural supernatant of METTL3-overexpressed cells (*n* = 3; ^∗∗^*P* < 0.01 and ^∗∗∗^*P* < 0.001).

As evidenced by the CCK-8 assay, the proliferation of macrophages was significantly inhibited in METTL3-overexpressed pTHP-1 cells after being activated by LPS for 12, 24, and 48 hrs (Figure 4(a)). In addition, the generation of IL-6 and TNF-*α* induced by LPS was obviously prevented when METTL3 was overexpressed in pTHP-1 macrophages (Figures 4(b) and 4(c)). Accordingly, as a key enzyme of N^6^-methyladenosine (m^6^A) methylation, METTL3 could affect RA by inhibiting the proliferation and inflammatory response in macrophages, which played a crucial role in RA.

### 3.5. METTL3 Inhibited the Inflammatory Response of pTHP-1 Macrophages through NF-*κ*B

**Figure 5:**
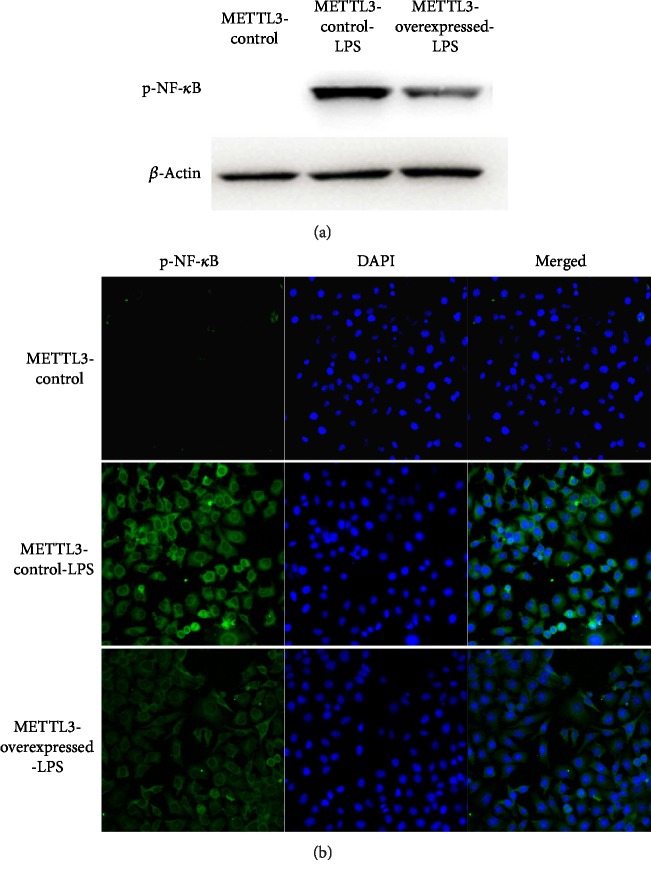
METTL3 attenuated LPS-induced inflammation in macrophages through the NF-*κ*B signaling pathway. (a) METTL3 inhibited the phosphorylation of NF-*κ*B in cells induced by LPS (*n* = 3). (b) METTL3 inhibited the nucleus translocation of p-NF-*κ*B in cells induced by LPS (*n* = 3).

Dysregulation of METTL3 seemed to influence the inflammation response of macrophages. Nonetheless, the potential molecular mechanism remained vague. In this study, we found that the phosphorylation of NF-*κ*B was obviously restrained in METTL3-overexpressed pTHP-1 cells although cells were activated by LPS for 4 hrs (Figure 5(a)). Besides, the nuclear translocation of phosphorylated NF-*κ*B in cells was also restrained when METTL3 was overexpressed (Figure 5(b)). Taken together, the modifying effects of METTL3 on LPS-induced inflammation in macrophages were dependent on the transcriptional factor NF-*κ*B.

## 4. Discussion

RA is a chronic and systemic autoimmune disease, the etiology of which is largely unknown [20]. It has been well documented that multiple factors are associated with inflammation and immune disorders in RA, including genetic factors, environmental factors, and epigenetic deregulation [21, 22]. Various inflammatory mediators are responsible for arthritis and articular deformity, such as TNF-*α*, IL-6, and IL-17 [23]. Apart from inflammation, autoimmune disorders are closely related to the development and progression of RA. As a key enzyme of m^6^A methylation modification, METTL3 has been suggested to regulate inflammation and autoimmune balance [13, 19]. The present study firstly shows the evidence that METTL3 is upregulated in RA and positively associated with the disease activity. Besides, METTL3 inhibits the proliferation and activation of macrophages through NF-*κ*B.

m^6^A is a methylation at the N6 position of adenosine, which is regarded as the most abundant epitranscriptomic modification of mRNA in eukaryotic cells [24]. A number of studies have demonstrated that m^6^A methylation is involved in various physiological processes including mRNA stability and translation. Its modifying effects on embryonic development, cell differentiation, and stress have been well demonstrated [24–26]. There are several key genes involved in m^6^A methylation modification, primarily including METTL3, METTL14, FTO, ALKBH5, and YTHDF [27, 28]. METTL3 is also known as MTA70, which is originally identified as a methyltransferase involved in the process of m^6^A methylation [29]. Accumulated evidence has strongly supported that METTL3 is involved in a variety of physiological contexts and in cancers [30–32]. METTL3 is located in a nuclear speckle, which reveals that METTL3 may play an important role in RNA metabolism [33, 34]. Given its effects on inflammation, cancer, and immune regulation [19], we hypothesize that METTL3 may affect the development and progression of RA by regulating macrophage-mediated inflammation. In this study, we firstly detected the expression of m^6^A methylation-associated genes (METTL3, FTO, ALKBH5, METTL14, YTHDF1, and YTHDF2) in PBMCs from RA patients and found that METTL3 was obviously upregulated in RA compared with healthy controls. Interestingly, LPS stimulation could enhance total m^6^A content of macrophages in a time-dependent manner by upregulating METTL3. It can be concluded that METTL3 is upregulated in macrophages under the circumstances of inflammation.

Macrophages are activated when they are infected by pathogenic factors such as LPS, PGN, and nucleotide compositions of pathogenic microorganisms, which leads to the phosphorylation and translocation of the transcriptional factor NF-*κ*B to the nucleus and induces the generation of target genes related to inflammation, for instance, IL-6 and TNF-*α* [33, 35, 36]. The NF-*κ*B signaling pathway is a classic pathway related to inflammation and immune regulation in RA [37]. Growing data showed that expression and regulation of m^6^A methylation-related genes are associated with a variety of immune signaling pathways, particularly the NF-*κ*B pathway [8, 18]. In this study, METTL3 has been found to be upregulated in peripheral blood monocytes, the critical cell type in PMBCs involved in inflammation and immune regulations. In addition, we have demonstrated the effect of METTL3 on m^6^A modification in macrophages stimulated by LPS. As a result, we hypothesize that METTL3-dependent m^6^A modification is associated with inflammation induced by LPS in macrophages. After verification in experiments in vitro, we have found that METTL3 could prevent macrophages from proliferation and production of inflammation-associated cytokines, namely, IL-6 and TNF-*α*. Moreover, its inhibitory effects on LPS-induced inflammation in macrophages were dependent on the NF-*κ*B signaling pathway. However, whether other key genes of this typical signaling pathway are involved in m^6^A modification in macrophages except for NF-*κ*B needs to be investigated by more future studies.

In summary, this study shows strong evidence supporting the vital role of METTL3 in RA. METTL3 can attenuate LPS-induced inflammation in macrophages through NF-*κ*B. The findings in this study are useful for understanding RA pathogenesis and exploring novel biomarkers for RA diagnosis and treatment.

## Figures and Tables

**Table 1:** Characteristics of participants.

	RA(*n* = 47)	Healthy controls (*n* = 30)	*P* value
Age (yrs, mean ± SD)	58.3 ± 6.1	49.9 ± 8.7	0.242
Gender (females/males)	34/13	21/9	0.105
Disease course (years)	16.6 ± 5.2	—	—
CRP (mg/l)	20.4 ± 3.5	—	—
ESR (mm/h)	41.0 ± 7.5	—	—
RF (IU/ml)	191.6 ± 5.7	—	—

## Data Availability

The data used to support the findings of this study are included within the article.
